# Resting-state functional connectivity alterations in post-stroke cognitive impairment: a systematic review

**DOI:** 10.1007/s11682-025-01013-w

**Published:** 2025-06-04

**Authors:** Elena Sagues, Francisco Alfaro, Ramón Ramos-Rodríguez, Natalia García-Casares

**Affiliations:** 1 Department of Neurology, University of Iowa Health Care, Iowa City, USA; 2 Departamento de Medicina. Facultad de Medicina, Universidad de Málaga, 29010 Bulevard Louis Pasteur, Málaga, Spain; 3 Centro de Investigaciones Médico-Sanitarias (CIMES), Universidad de Málaga, Málaga, Spain; 4 Instituto de investigación Biomédica y plataforma en Nanomedicina, IBIMA-Plataforma BIONAND, Málaga, Spain

**Keywords:** Functional magnetic resonance, Functional connectivity, Resting-state, Post-stroke cognitive impairment, Networks

## Abstract

Post-stroke cognitive impairment is a common and highly disabling multi-domain cognitive decline with unclear pathophysiological mechanisms. Its extent may be predicted by functional neuroimaging techniques, which could enhance our understanding of its underlying pathophysiology. Synthesize the state-of-the-art knowledge on resting-state functional magnetic resonance imaging findings in post-stroke cognitive impairment, their correlation with cognitive performance and the secondary compensatory connectivity changes. We performed a qualitative systematic literature review of reported data of resting-state functional magnetic resonance imaging abnormalities in post-stroke cognitive impairment, consulting the electronic databases Medline, Scopus, Web of Science, Cochrane, and BASE until June 2024. We selected 23 studies. They reported several key brain areas and network abnormalities associated with post-stroke cognitive impairment compared to controls and non-demented patients. The main areas with decreased functional connectivity were the medial prefrontal cortex, the inferior frontal gyrus, the posterior and anterior cingulate cortex, the precuneus and the hippocampus. The principal network altered was the default mode network, which includes the majority of the brain structures previously mentioned. Additionally, increased functional connectivity in other contralesionally areas may represent maladaptive or compensatory changes post-stroke. Resting-state functional magnetic resonance imaging is a promising tool for studying functional changes associated with post-stroke cognitive impairment. It may enhance our understanding of its pathophysiology and improve selection of suitable period or candidates for cognitive rehabilitation.

**Supplementary Information:**

The online version contains supplementary material available at 10.1007/s11682-025-01013-w.

## Introduction

Post-stroke cognitive impairment (PSCI) is considered a disabling multi-domain cognitive decline manifested in the first year after the stroke, which estimated prevalence ranges from 17.5% to 54.9%, (Sexton et al., 2019). A precise pathophysiological explanation has not been yet confirmed, being several mechanisms proposed as the necrosis of brain tissue (Ferrarini et al., 2014), abnormal neurotransmitters production (Gabrieli, 1996), secondary white matter demyelination and beta-amyloid deposition after ischemia (Wu et al., 2008). The stroke characteristics, the presence of concomitant lesions in time and place and the compensatory capacity of the nervous system circuits are known predictors of cognitive deterioration (Pendlebury & Rothwell, 2009). Structural imaging studies have previously identified strategic areas whose damage or atrophy post-stroke may predispose to cognitive declines, such as the left angular gyrus, the left basal ganglia and the white matter around it (L. Zhao et al., 2018).

In recent years, resting-state functional magnetic resonance imaging (fMRI) has been demonstrated to be a useful technique to investigate the functional architecture of the human brain using fluctuations in low-frequency blood oxygenation level-dependent (BOLD) signals. More efficiently organized brain networks have been associated with better cognitive performance (Van Den Heuvel et al., 2009). This technique appears to be superior to structural ones to predict visual and verbal memory (Siegel et al., 2016), as stroke-induced lesions not only affect the functionality of the lesion site but may impact interactions among areas distant to it (Grefkes & Fink, 2011). Additionally, these functional alterations may be an early marker of underlying disease before macrostructural changes occur (van Norden et al., 2012).

Evidence regarding the functional connectivity (FC) of specific brain areas and networks altered in PSCI in fMRI is slightly heterogeneous in terms of the population selection criteria, the type of strokes included and the postprocessing protocols performed. This systematic review synthesizes the state-of-the-art knowledge on fMRI findings in PSCI, their correlation with cognitive performance classified by domains and the potential secondary FC compensatory changes. Increasing evidence in this field will help facilitate further understanding of PSCI pathogenesis. Moreover, machine learning algorithms and predictive models using fMRI resting-state sequences post-stroke have already demonstrated accurate prediction of cognitive outcomes in the long term (Kliper et al., 2016; Lopes et al., 2021; Siegel et al., 2016; Y. Zhao et al., 2023). Therefore, additional research in this field could find potential functional neuroimaging biomarkers that will allow the identification of populations at increased risk of PSCI that could benefit from early cognitive rehabilitation.

## Methods

This systematic review has been conducted and authored according to the Preferred Reporting Items for Systematic Reviews and Meta-Analyses (PRISMA) reporting guidelines updated in 2020 (Page et al., 2021).

### Eligibility criteria

The inclusion criteria for this review encompassed articles published in peer-reviewed journals that presented FC data from resting-state fMRI in human populations undergoing cognitive assessment. Post-stroke cognitive deficits had to be assessed using a standardized cognitive evaluation tool. Studies employing task-based fMRI or other modalities to study connectivity, such as transcranial magnetic stimulation, were excluded to mitigate complexity arising from differing experimental paradigms and data analysis techniques. Similarly, case reports, systematic or literature reviews, letters to the editor, and conference abstracts were also excluded.

### Information sources

We consulted the electronic databases PubMed/Medline, Scopus/Embase, Web of Science, and BASE academic search engine without any time restrictions. Additional sources were bibliographic references of the included studies.

### Search strategy

Two independent investigators performed a systematic review of the literature with the keywords “Post-stroke cognitive impairment, post-stroke dementia, functional magnetic resonance, connectivity, default mode network and resting state, low-frequency fluctuations, regional homogeneity” and the search code: (post-stroke cognitive impairment OR post-stroke dementia) AND (functional magnetic resonance OR connectivity OR default mode network OR resting state OR low-frequency fluctuations OR regional homogeneity).

### Selection process

Two independent reviewers without any automatization tool evaluated the studies to assess their eligibility. A full-text evaluation was performed, and the ROBINS-I scale (Risk of Bias in Non-randomized Studies of Interventions) was used to assess the quality of the included studies (Supplementary Table 1).

### Data collection and data items

Data was manually collected in a Word table by two independent reviewers without any automatization tool. We extracted the following items: first author, year of publication, study design, sample size, average age, groups, type of stroke in terms of location, laterality and type (ischemic or hemorrhagic), the time from the event to the fMRI realization, the cognitive evaluations performed, the fMRI preprocessing and postprocessing. Outcomes variables were the alterations on the fMRI significantly found in PSCI or correlated with cognitive performance.

## Results

### Studies selection

**Fig. 1:**
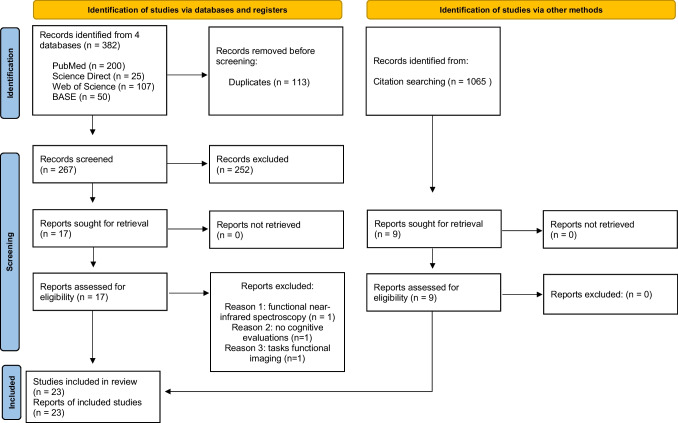
Flow chart of the studies selection. The identification, screening, and inclusion phases of the process are represented by the number of studies at each stage

The initial search generated a total of 382 records: 200 from PubMed/Medline, 25 from Scopus/Embase, 107 from Web of Science and 50 from the BASE search engine. No record was obtained in Cochrane. After removing duplicates and screening the results we selected 17 articles to assess for eligibility. Additionally, 9 more articles were obtained from bibliographic references. After a full text read, 3 articles were excluded, one of them because they used functional near-infrared spectroscopy instead of fMRI (Yongmei and Peng 2022), another because it did not perform cognitive evaluations post-stroke (Jiang et al., 2018), and the last one because performed fMRI with tasks (Snaphaan et al., 2009). The most common quality concern after assessing articles using ROBINS-I scale was the non-blinding of the person administering the MoCA in post-stroke patients and controls, which commonly occurs if the exam is performed at discharge or by the same neurologist following up with the patient after a stroke. We included a total number of 23 studies to include in our review (Bournonville et al., 2018; Cai et al., 2021; R. Dacosta-Aguayo et al., 2014; Rosalia Dacosta-Aguayo et al., 2015; Ding et al., 2014; Jung et al., 2021; Kliper et al., 2016; Jiao Liu et al., 2017; Jingchun Liu et al., 2014; Lopes et al., 2021; Miao et al., 2021; Min et al., 2022; Park et al., 2014; Peng et al., 2016; Siegel et al., 2016; Snaphaan et al., 2009; Vicentini et al., 2021; Wang et al., 2022; Xu, 2022; Yue et al., 2023; Zhang et al., 2020; Y. Zhao et al., 2023; Z. Zhao et al., 2021; Zhu et al., 2022). Figure 1 contains the flow diagram of the study's selection.

### Studies characteristics

Table 1 summarizes the 23 studies that proved eligible for inclusion, their characteristics, and the results obtained. The sample size ranged from 22 to 122. The average ages ranged from 50 to 69 years old. The authors either selected post-stroke patients at a certain time lapse and classified them as cognitively impaired or demented and non-cognitively impaired, to compare the fMRI alterations in both groups (Bournonville et al., 2018; Cai et al., 2021; R. Dacosta-Aguayo et al., 2014; Ding et al., 2014; Min et al., 2022; Peng et al., 2016; Zhang et al., 2020; Z. Zhao et al., 2021; Zhu et al., 2022); or performed cognitive evaluations in post-stroke patients (Rosalia Dacosta-Aguayo et al., 2015; Kliper et al., 2016; Jingchun Liu et al., 2014; Lopes et al., 2021; Park et al., 2014; Siegel et al., 2016; Snaphaan et al., 2009; Vicentini et al., 2021). Alternatively, they just included PSCI patients (Jung et al., 2021; Jiao Liu et al., 2017; Miao et al., 2021; Wang et al., 2022; Xu, 2022; Yue et al., 2023; Y. Zhao et al., 2023). The inclusion criteria of most of the studies were right-handed patients with their first-ever stroke that were not diagnosed previously with dementia. They all excluded those patients with MRI contraindications or with severe aphasia that made impossible the cognitive evaluation. Regarding the type of stroke included, most of the authors when comparing PSCI and non-PSCI, used groups that presented similar average stroke volume, lesion number and laterality proportions. They specified in which period the fMRI was obtained, being in their majority in the subacute period (before 3–6 months depending on the criteria). Only 3 studies (Jiao Liu et al., 2017; Jingchun Liu et al., 2014; Y. Zhao et al., 2023) studied the chronic phase (> 12 months) attempting to describe the permanent damage post-stroke. Additionally, 2 studies performed fMRI in two different periods, acute and subacute (Zhang et al., 2020) and subacute and chronic (Vicentini et al., 2021), to assess the brain changes in this time course. Most of the authors included different vascular territories and lateralities in their populations.

**Table 1:** Characteristics and results of the studies

Authors and year	Sample size	Mean age (years)	Study design	Stroke (location/vascular territory, type and time from event)	Cognitive tests	Networks	Results	Conclusions
Ding X et al. 2014	N = 40	64	L Groups: C (N = 20) PSCI (N = 11) Non-PSCI (N = 9)	• Carotid and VB • Ischemic • 5–10 days	MoCA MMSE	DMN	Non-PSCI had significantly increased FC in mPFC and HPC than PSCI (p < 0.05) FC in PCC/PCu correlated to the MoCA score at 10 th day (r = 0.46, p = 0.002) and FC in left HPC correlated with the MoCA score at 3 months (r = 0.51, p < 0.001)	PSCI have decreased FC in mPFC and HPC than Non-PSCI and FC in PCC/PCu and left HPC correlated with cognitive scores
Park J et al. 2014	N = 22	58	L Groups: C (N = 11) S (N = 11)	• Right • 1, 3 and 6 months	MMSE DST-F/B VST-F/B RCFT-DR TMT-A/B	DMN	Right mFG FC was significantly correlated with RCFT-DR score at 3 months (r = 0.775,P < 0.041) and 6 months (r = 0.821, p < 0.023) Patients with decreased FC in the PCC and PCu at 6 months were associated with persistent PCSI	Decreased PCC/PCu FC at 6 months was associated with persistent PSCI, whereas right mFG FC correlated with its recovery
Liu J et al. 2014	N = 38	56	CS Groups: C (N = 20) S (N = 18)	• Subcortical • > 6 months (chronic)	MoCA	DMN	ReHo of PCC was positively correlated with MoCA scores post-stroke (r = 0.51, p = 0.046)	PCC FC was correlated with MoCA score post-stroke
Liu J et al. 2017	N = 54	58	CS Groups: C (N = 27) PMD (N = 27)	• Right • Over 12 months	MMSE WMS-CR	DMN and DAN	FC in right precentral, right mFG, right inferior FG, right insula and left ACC correlated with WMS scores (p < 0.001)	FC in right precentral, mFG, inferior FG, insula and left ACC correlated with WMS scores post-stroke
Dacosta-Aguayo R et al. 2014	N = 36	63	L Groups: C (N = 18) PSCI (N = 10) Non-PSCI (N = 8)	• ACA, MCA and PCA • Ischemic • 3 months	MMSE MoCA DST-F/B TMT-A/B BNT	DMN, BGN, FN, FTN, SVN and PN	Non-PSCI had increased activity in DMN and FTN and decreased in BGN compared to PSCI. In PSCI, BGN activity was negatively correlated with TMT-A score, whereas FN activity was positively correlated with TMT-A score	Non-PSCI had increased DMN and FTN activity than PSCI, and FN activity correlated with TMT-A score in PSCI
Dacosta-Aguayo R et al. 2015	N = 28	63	L Groups: C (N = 17) S (N = 11)	• Cortical and subcortical • Right • Ischemic • 3 months	MMSE MoCA FBI GPT IQ IQCODE TMT-A	DMN	FC in FG, PCC, right PL and left PhG positively correlated with semantic fluency (r = 0.454, p = 0.023), phonetic fluency (r = 0.523, p = 0.007) and MMSE (r = 0.528, p = 0.007)	FC in FG, PCC, right PL and left PhG correlated with cognitive performance post-stroke
Siegel JS et al. 2016	N = 132	53	CS Groups: S (N = 132)	• Over 1–2 weeks	Visual attention, memory and language evaluation	CON, VAN and DMN	Visual and verbal memory were predicted with fMRI data using machine learning and decreased interhemispheric integration and interhemispheric segregation strongly related to behavioral impairment	fMRI predicts memory impairment post-stroke
Peng C et al. 2016	N = 62	58	CS Groups: C (N = 30) PSCI (N = 16) Non-PSCI (N = 16)	• Ischemic • Subcortical • MCA • > 3 months	MMSE MoCA AVLT SDMT CFT FDST DST-F/B	-	PSCI had decreased ReHo in ACC and left PCC/Pcu compared to Non-PSCI; being ReHo in ACC correlated with SDMT and CFT scores (r = 0.399,p = 0.036; r = 0.397, p = 0.036) and ReHo in the left PCC/PCu correlated with DST-F scores (r = 0.485, p = 0.009)	ACC and PCC/PCu decreased rs FC is associated with PSCI and correlates to cognitive scores
Kliper E et al. 2016	N = 83	69	L Groups: S (N = 83)	• Ischemic • Over 1 week	Global cognitive score by computer testing	-	HPC FC was correlated with cognitive state 6 and 12 months post-stroke (r = 0.274, p = 0.05 and r = 0.281, p = 0.05)	HPC FC helps predict CI post-stroke
Bournonville C et al. 2018	N = 56	63	CS Groups: PSCI (N = 29) Non-PSCI (N = 27)	• Ischemic • 6 months	IQCODE	-	Connection strengths were weaker in patients with PSCI (p < 0.05) but global topological organization did not differ	PSCI presents a deficit of the FC of a whole brain network
Lopes R et al. 2021	N = 72	-	L Groups: S (N = 72)	• 6 months	IQCODE	-	A machine learning model was able to predict at 6 months post-stroke the memory, attention, visuospatial and language functions at 36 months (r = 0.67, 0.73, 0.55 and 0.48, respectively)	DMN analysis helps predict CI post-stroke
Zhang J et al. 2020	N = 47	50	L Groups: C (N = 28) PSCI (N = 11) Non-PSCI (N = 8)	• Subcortical • Over 10 days and at 3 months	MoCA-Beijing	-	Non-PSCI had extra compensatory paths between baseline and 3 month post-stroke Extra paths from the contralesional ventralanterior nucleus of the thalamus FC strength correlated with cognitive scores post-stroke	Prefrontal-basal ganglia circuit FC post-stroke may predict better compensation and cognitive outcomes
Vicentini JE et al. 2021	N = 57	-	L Groups: C (N = 20) S (N = 37)	• Ischemic • Over 1 month and at 6 months	MoCA	DMN, SN and CEN	Better cognitive performance post-stroke was associated with stronger interhemispheric and ipsilesional DMN FC, and weaker contralesional SN FC	Ipsi and contralesional DMN alterations associates with CI
Miao G et al. 2022	N = 58	58	CS Groups: C (N = 21) Isc PSCI (N = 21) Hem PSCI (N = 16)	• 1 week to 3 months	MoCA MMSE	DMN, SN, CN and ORB	PSCI had significantly decreased feeder and local connections on SN, DMN, CN and ORB, mainly in caudate, compared to controls SMN and VN connections in hem PSCI, and SN and DMN in isc PSCI, were positively correlated to the MMSE and MoCA scores	Disruption of the local organization but preserved core networks were significantly found in PSCI
Rao B et al. 2022	N = 58	58	CS Groups: C (N = 21) Isc PSCI (N = 21) Hem PSCI (N = 16)	• Over 3 months	MoCA MMSE	DMN, AN, VN, SMN, ON, PN, FPN, ECN, SN and CN	PSCI showed dysfunctional FC within the primary networks and between the primary and high-order cognitive control domains (p < 0.01)	PSCI had decreased FC in primary and high order cognitive networks
Zhao Z et al. 2021	N = 61	64	CS Groups: C (N = 17) PSD (N = 20) PSND (N = 24)	• Ischemic • Over 6 months	IQCODE MMSE MiniCog	-	PSD had significantly decreased inward and increased outward flow within bilateral HPC/HPC head and left HPC body compared to PSND/C (p < 0.05) Cognition scores correlated with FC of bilateral HPC and left HPC head in PSD (p < 0.05)	HPC alterations in transmission and reception of information are present in PSD and correlate with cognitive scores
Jung J et al. 2021	N = 49	62	CS Groups: C (N = 16) PSCI (N = 33)	• Ischemic • Over 3 months	MoCA BCoS	-	Decreased FC between HPC and inferior PL was found in PSCI compared to C, being associated with impaired memory function However, the FC of HPC subfields with cerebellum was increased in PSCI compared to C	Decreased HPC-inferior parietal lobe FC is associated with PSCI
Cai H et al. 2021	N = 65	63	CS Groups: C (N = 21) PSD (N = 20) PSND (N = 24)	• Ischemic • Over 6 months	MMSE Minicog	-	PSD had lower fALFF in the right inferior FG than both PSND and C, not being such differences observed between PSND and C	PSD present specific functional alteration patterns, especially in the right inferior frontal gyrus
Wang S et al. 2022	N = 74	58	CS Groups: C (N = 37) PSCI (N = 37)	• Over 3 months	MoCA MMSE	-	PSCI had decreased FC in left language regions (angular gyrus, temporal pole, inferior temporal gyrus and caudate) and compensational increased FC in right middle FG, HPC, Cu and PCu	PSCI patients had decreased FC in the left language related brain regions
Min Y et al. 2022	N = 64	51	CS Groups: C (N = 29) PSCI (N = 20) Non-PSCI (N = 15)	• Subcortical • Over 10 days	MoCA AVLT RCFT-DR SDMT BNT AFT TMT-A/B	-	Right PhG had significantly increased FC in PSCI and Non-PSCI and showed a negative correlation with MoCA, AVLT, RCFT-DR, SDMT, BNT and AFT; and a positive correlation with TMT-A and TMT-B	Right PhG FC was significantly increased in PSCI and Non-PSCI and negatively correlated with cognitive scores
Zhu H et al. 2022	N = 69	52	CS Groups: C (N = 29) PSCI (N = 23) Non-PSCI (N = 17)	• Ischemic • BG region • Over 10–14 days	MoCA-Beijing AVLT RCFT-DR RCFT-V AFT BNT SDMT TMT-A/B CWT	Multiple	PSCI exhibited reduced FC comprising intra-frontal (19.3%), frontal-limbic (13.6%), and frontal-parietal (10.2%) networks	PSCI had significantly reduced FC in FPN and CON
Zhao Y et al. 2023	N = 122	-	CS Groups: C (N = 52) PSCI (N = 70)	• Chronic (> 12 months)	PALPA battery	-	FC profiles predicted phonology (p = 0.013), semantics (p = 0.001) and fluency (p = 0.005) but not executive function	FC models predict behavioral deficits post-stroke
Yue X et al. 2023	N = 41	54	CS Groups: C (N = 24) PSCI (N = 17)	• Ischemic • Cortical and subcortical • 1 week to 3 months	MMSE	AN, left ECN, LN, PCu, right ECN, SN, VN, dorsal DMN, ventral DMN, PVN, SVN	PSCI exhibited significantly reduced dorsal DMN, VN and AN FC (p < 0.05)	PSCI had significantly reduced FC in DMN, VN and AN

*ACC* Anterior cerebral artery, *ACC* anterior cingulate cortex, *AFT* Animal Fluency Test, *AN* Auditory network, *AVLT* Auditory Verbal Learning Test, *B-coS* Birmingham cognitive screen, *BNT* Boston Naming Test, *BG* Basal ganglia, *BGN* Basal ganglia network, *C* controls, *Cu* Cuneus, *CBT* corsi block-tapping, *CDT* clock drawing test, *CEN* central executive networks, *CFT*, complex figure test, *CN* cerebellar network, *CS* cross-sectional, *CON* cingulo-opercular network, *CWT* Color-Word Test-Chinese version, *DAN* dorsal attention network, *DMN* Default mode network, *DST-B* Backward digit span test, *DST-F* forward digit span test, *ECN* executive control network, *fALFF* Fractional amplitude of low-frequency fluctuations, *FBI* Frontal behavioral inventory, *FC* functional connectivity, *FG* fronta gyrus, *FN* frontal network, *FPN* fronto-parietal network, *FTN* fronto-temporal network, *GPT* Grooved pegboard test, *Hem PSCI* post hemorrhagic stroke cognitive impairment, *HPC* hippocampus, *HVLT* Hopkins Verbal Learning Test, *IQ* Intelligence quotient, *IQCODE* Informant questionnaire on cognitive decline in elderly, *Isc PSCI* post ischemic stroke cognitive impairment, *L* longitudinal, *MCA* medial cerebral artery, *mFG* Middle frontal gyrus, *MMSE*, minimental scale examination, *MoCA* Montreal cognitive assessment, *MoCA-Beijing* Montreal Cognitive Assessment Beijing version, *mFC*, medial frontal cortex, *mPFC* medial prefrontal cortex, *Non-PSCI* post-stroke without cognitive impairment, *ON* occipital network, *ORB* orbitofrontal cortex, *PCA* posterior cerebral artery, *PCC* posterior cingulate cortex, *PCu* precuneus, *PhG* parahippocampal, *PMD* Post-stroke memory dysfunction, *PN* Parietal network, *PL* parietal lobe, *PSCI* post-stroke cognitive impairment, *PVN* Primary visual network, *RCFT-DR* Rey-Osterrieth Complex Figure Test-Delayed Recall, *RCFT-V* Rey-Osterrieth Complex Figure Test Copy for visuospatial ability, *ReHo* regional homogeneity, *rsFC* resting-state functional connectivity, *S* stroke, *SDMT*, Symbol Digit Modality Test, *S-IQCODE* short informant questionnaire on cognitive decline in the elderly, *SMN* sensory motor network, *SN* salience network, *SVN* Secondary visual network, *TMT-A/B* Trail Making Test A/B parts, *TL* temporal lobe, *VB* Vertebrobasilar, *VAN* Ventral attention network, *VN* visual network, *VST-B* Backward visual span test, *VST-F* forward visual span test, *WMS-CR* Wechsler Memory Scale-Chinese revision, *WMS* Wechsler Memory Scale

In relation to the fMRI preprocessing, all the studies analyzed the resting state FC postprocessing, brain functional networks were frequently studied, especially the default mode network (DMN) (R. Dacosta-Aguayo et al., 2014; Rosalia Dacosta-Aguayo et al., 2015; Ding et al., 2014; Jiao Liu et al., 2017; Jingchun Liu et al., 2014; Lopes et al., 2021; Miao et al., 2021; Min et al., 2022; Park et al., 2014; Siegel et al., 2016; Vicentini et al., 2021; Wang et al., 2022; Yue et al., 2023). Other networks analyzed were the salience network (Miao et al., 2021; Vicentini et al., 2021; Yue et al., 2023), the dorsal attention network (Jiao Liu et al., 2017), the central executive network (Vicentini et al., 2021; Yue et al., 2023), the cerebellar network (Miao et al., 2021), etc. The software tools used are diverse, with examples including SPM (Statistical Parametric Mapping) (Zhao Y et al. 2023, Zhang J et al. 2020), GIFT (Group independent component analysis of fMRI Toolbox) (Yue et al., 2023), FSL (FMRIB Software Library) (Wang Y et al. 2013), and analysis parameters vary significantly among authors. We acknowledge that this limitation may affect the comparability of results across research. Instead, it may be more beneficial to focus on identifying common brain regions implicated in PSCI and comparing their topographical results. Finally, we identified the cognitive evaluations performed in each study, which in their majority consisted of the Montreal Cognitive Assessment (MoCA) and the Mini-mental State Evaluation (MMSE). On two occasions (Cai et al., 2021; Z. Zhao et al., 2021) the miniCog evaluation was used instead of the MoCA to correct for illiterate and low-educated elderly patients.

### Results of the studies

Resting-state fMRI studies have found decreased FC in several specific brain areas and abnormalities on brain networks significantly associated with PSCI and in correlation with cognitive test scores**.** Other areas that were inversely hyper-functional in PSCI may represent maladaptive or compensatory brain changes, as they were frequently correlated with worse cognitive outcomes.

#### Brain areas with decreased FC in fMRI in PSCI

Compared to patients without PSCI, the FC of several brain areas was significantly reduced in PSCI, including the medial prefrontal cortex (Ding et al., 2014), right inferior frontal gyrus (Cai et al., 2021), hippocampus (Ding et al., 2014; Kliper et al., 2016; Z. Zhao et al., 2021), posterior cingulate cortex (Park et al., 2014; Peng et al., 2016), precuneus (Park et al., 2014; Peng et al., 2016), anterior cingulate cortex (Peng et al., 2016) and left language-specific areas like the angular gyrus, temporal pole, inferior temporal gyrus and caudate (Wang et al., 2022). When the hippocampus FC in post-stroke patients was explored in depth, dementia-specific changes consisting of significantly decreased inward and increased outward information flow between the hippocampal head, body and several cortical regions, as the parietal lobe, were found (Jung et al., 2021; Z. Zhao et al., 2021). Contrarily, the same authors found increased FC between the hippocampus and the cerebellum in PSCI compared to controls. Finally, a model comparing FC in each cognitive domain region of interest in chronic PSCI patients and healthy subjects predicted phonology, semantics and fluency, but not executive function (Y. Zhao et al., 2023).

#### FC correlation with cognitive tests and domains

PSCI involves numerous domains, most commonly memory, attention and executive functions together with reduced reaction and information processing speeds. Several patterns of specific node alterations in relationship with a decline in each domain were described by Bournonville et al. (Bournonville et al., 2018). In broad terms, the main functional disconnections were between the superior, middle, and inferior frontal gyri and the superior and inferior temporal gyri. Most of the studies did not perform multi-domain assessments but selected a specific cognitive evaluation test. Most of them used the MoCA that measures attention, concentration, executive functions, memory, language, visuospatial skills, abstraction, calculation and orientation. Decreased FC in the precuneus (Ding et al., 2014), left hippocampus (Ding et al., 2014) and posterior cingulate cortex (Jingchun Liu et al., 2014) was correlated with decreased MoCA scores. The Rey Complex Figure Task score post-stroke, which measures the visual-constructional ability and memory, positively correlated with the medial frontal gyrus correlated FC (Park et al., 2014). Wechsler memory scale assesses different memory functions, FC in the right precentral, right medial frontal gyri, right middle frontal, right inferior frontal gyri, right insula and left anterior cingulate correlated with increased scores post-stroke (Jiao Liu et al., 2017). The Trail Making Test part A measures the processing speed, in PSCI, the frontal network brain activity was positively correlated with its score (R. Dacosta-Aguayo et al., 2014). Semantic and phonetic fluency are also included in the executive functions evaluation, FC in the frontal gyrus, posterior cingulate gyrus, right parietal lobe and left parahippocampus correlated with their scores, as well as with MMSE results in post-stroke patients (Rosalia Dacosta-Aguayo et al., 2015). The Symbol Digit Modalities test measures information processing speed and the Complex Figure test evaluates cognitive flexibility, and FC in the anterior cingulate cortex correlated with both test scores (Peng et al., 2016). Finally, the forward Digit Span test measures working memory capacity, FC in the left posterior cingulate cortex and precuneus correlated with its results (Peng et al., 2016).

#### Brain networks alterations in PSCI

PSCI patients, despite conserving the topological brain functional network organization (Bournonville et al., 2018), showed a general functional disconnection within the primary networks (auditory, sensorimotor and visual networks) and between the primary and high-order cognitive control domains (Xu, 2022). Specifically, altered DMN FC (R. Dacosta-Aguayo et al., 2014; Ding et al., 2014; Miao et al., 2021; Vicentini et al., 2021) was significantly associated with PSCI and negatively correlated with cognitive scores. Decreased feeder and local connections on the cerebellar and orbitofrontal networks were also significantly found in PSCI (Miao et al., 2021; Zhu et al., 2022). Whereas, non-PSCI had increased brain activity in the frontotemporal network than PSCI (R. Dacosta-Aguayo et al., 2014; Zhu et al., 2022). Sensory-motor and visual networks were correlated with MoCA scores post-stroke (Miao et al., 2021). Contrarily, contralesional salience network FC negatively correlated with MoCA scores (Miao et al., 2021; Vicentini et al., 2021). Finally, Siegel et al. (Siegel et al., 2016) applied a machine learning algorithm to predict memory function post-stroke using FC in DMN, ventral attention and cingulo-opercular networks. They concluded that altered interhemispheric connections across all brain areas were associated with behavioral impairment and general cognitive deficits.

#### Post-stroke secondary compensatory connectivity changes

Some authors reported in the subacute period post-stroke increased FC in the ipsihemisphere and contrahemisphere that may represent compensatory changes. Wang S et al. (Wang et al., 2022) described in left-stroke patients enhanced processing and relaying of information of some right high-order cognitive brain regions such as the middle frontal gyrus, hippocampus, cuneus, and precuneus that may compensate for the left-side damage. Finally, Min et al. (Min et al., 2022) observed that right parahippocampal FC was significantly increased in PSCI and non-PSCI and was negatively correlated with cognitive functions in the acute post-stroke period, which may indicate brain node reorganization.

Several studies performed longitudinal follow-ups of their patients to study the FC changes over the months following an stroke in relationship with their cognitive deterioration. Park J et al. (Park et al., 2014) performed fMRI studies at one, three and six months post-stroke, finding decreased DMN FC in the posterior cingulate cortex, precuneus and medial frontal gyrus in the first months that gradually resolved at 3 months, suggesting that this is the critical period for neural reorganization. Those with persistently decreased FC at 6 months in the posterior cingulate cortex and precuneus were significantly associated with chronic cognitive deficits. Zhang J et al. (Zhang et al., 2020) described extra compensatory paths between baseline and 3-month post-stroke in non-PSCI patients, finding a correlation between the FC of the extra paths from the contralesional ventral anterior nucleus of the thalamus and cognitive scores post-stroke.

## Discussion

In this systematic review, we examine the fMRI correlates of PSCI across the 23 included studies. Through this discussion, we aim to deepen the understanding of FC changes and their implications for cognitive recovery following stroke. Additionally, we aim to assist future studies in addressing the limitations identified in previous research.

Decreased resting-state FC in specific brain regions as the medial prefrontal cortex, the inferior frontal gyrus, the posterior and anterior cingulate cortex, the precuneus and the hippocampus may play an important role in the pathogenesis of PSCI, while increased FC in areas contralateral to the infarct may act as maladaptive or compensatory changes. Reduced FC in frontal lobe regions correlates with scores on neuropsychological tests assessing executive function, while areas involved in memory circuits, like the precuneus and insula, show decreased FC related to Wechsler Memory Scale scores. However, most studies primarily rely on MoCA and MMSE assessments for evaluating cognition. Future research should aim to establish more detailed associations using cognitive domain-specific tests.

Marked heterogeneity exists in the types of strokes included and the fMRI protocols used across the studies. In many cases, the area or type of stroke was not considered when analyzing the data. This may be justified by the hypothesis that a stroke can induce alterations in distant regions, affecting overall brain network functionality. However, studies using selective infarct locations may elucidate more information about the pathophysiology of PSCI. For example, Fan L et al. (Fan et al., 2019) study, which was not included in the review as they did not perform cognitive evaluations in their stroke patients, studied fMRI images just in cerebellar infarctions, finding significant FC alterations in the left hippocampus and right cingulate gyrus that may explain the cognitive alterations very frequently suffered in cerebellar strokes.

Previous studies associating brain areas with cognitive deficits mainly focuses on the role of acute and subacute CT and MRI. Several systematic reviews and meta-analyses concluded that brain atrophy and white matter hyperintensities in the medial temporal lobe were significantly correlated with PSCI (Ball et al., 2022a, b; Ball et al., 2022a, b; Casolla et al., 2019; F. Wang et al., 2021). Other key areas were the thalamus, cingulate gyrus, right superior temporal gyrus, right inferior parietal lobe, left middle occipital gyrus, right caudate and the cerebellum (Ahn et al., 2019; Stebbins et al., 2008; Yang et al., 2018). Previous studies using positron emission tomography (PET) with flutemetamol (18 F) in PSCI patients found tracer uptakes areas in the ipsilesional and contralesional hemispheres positively associated with cognitive performance, which may indicate their potential compensatory role (Huang et al., 2020). Nevertheless, functional imaging techniques appeared to be superior to structural imaging and lesion topography to predict visual and verbal memory post-stroke (Siegel et al., 2016). This may be explained by the general network disturbances secondary to FC alterations of remote areas from the original lesion, which may appear earlier compared to macro-structural changes. For instance, Bournonville et al. (Bournonville et al., 2018) found a generalized brain functional disconnection in PSCI compared to non-PSCI patients despite being in both groups the overall gray matter thickness and ischemic infarct topography similar. Kliper et al. studied the hippocampus combining diffusion tensor imaging and volumetric analyses alongside fMRI using a machine learning predictive model (Kliper et al., 2016). Combining the three techniques in future studies can offer a more comprehensive perspective on multimodal neuroimaging.

The location and extent of a brain infarct can significantly impact cognitive outcomes by disrupting several neural networks. Understanding these relationships may help clinicians predict and manage cognitive impairments in stroke survivors. Recent studies in lesion-deficit mapping have shown promising results, with functional lesion network mapping proving most effective in predicting language deficits (Siegel et al., 2016; Bowren JM et al. 2022). For instance, expressive language deficits correlate strongly with lesions in the left anterior insula, left frontal operculum, and left arcuate fasciculus. Lesions impacting receptive language are often found in the left superior longitudinal fasciculus, left frontal aslant tract, left parietal operculum, and left posterior insula. Anterograde verbal memory deficits tend to associate with lesions in the left fusiform and parahippocampal cortices, deep left frontal white matter, and left sub-insula/claustrum. Similarly, machine learning models hold significant potential for advancing our understanding and management of PSCI by integrating multimodal imaging data with clinical information. These models can be employed to predict early PSCI and guide personalized rehabilitation strategies. Several studies have demonstrated accurate long-term predictions of cognitive outcomes using machine learning (Kliper et al., 2016; Lopes et al., 2021; Siegel et al., 2016; Y. Zhao et al., 2023). Lopes et al. was able to predict memory, attention, visuospatial and language functions 36 months post-stroke using machine learning, finding specific patterns of FC for each of the four cognitive domains (Lopes et al., 2021). To optimize machine learning models, researchers should focus on enhancing training data quality, selecting appropriate algorithms suited for specific tasks, and establishing robust metrics for evaluating model performance.

The DMN was the primary network altered in PSCI, which includes the majority of the brain structures previously mentioned. The DMN is a set of widely distributed brain regions in the parietal, temporal and frontal cortex and it is of the most prominent resting state functional networks dominated by internally directed self-referential cognitive processes. Its decreased FC has been associated with poor cognition (Y. Wang et al., 2013) and reported in many different pathologies such as depression (Borserio et al., 2021), epilepsy (Parsons et al., 2020) and autism (Harikumar et al., 2021). On the contrary, the salience network is involved in the maintenance of homeostasis between internal and external stimuli and its increased FC has been associated with poor cognitive processing (Balthazar et al., 2014). Additionally, many other significant differences in resting activity were found between groups in other brain networks. Current research is working hard in consolidating evidence and creating PSCI networks-based maps to better understand changes post-stroke.

There is an ongoing debate in the literature regarding the role of areas with increased FC that negatively correlate with post-stroke cognitive performance. These regions may be activated as either compensatory mechanisms or maladaptive recovery processes following a stroke. In this review, these regions have predominantly involved contralesional areas, including the ventral anterior nucleus of the thalamus (Zhang et al., 2020), right high-order cognitive brain regions such as the middle frontal gyrus, hippocampus, cuneus, and precuneus (Wang et al., 2022) and right parahippocampus (Min et al., 2022). After a stroke, there is often increased activation in the brain areas surrounding the lesion. These perilesional areas may take over some functions of the damaged regions. Moreover, the contralesional hemisphere may also exhibit increased FC. Cortical neurophysiology is known to change over time from the acute/subacute period to the chronic stage after stroke, being this first interval the most critical period for brain reorganization (Park et al., 2014). Compensatory brain responses include the activation of areas normally connected to the injured site through a distributed network that may affect the contralesional hemisphere or the ipsilesional surrounding areas, depending on the cortical centers affected, the brain reserve and the lesion load (Umarova, 2017). For stroke patients, the premorbid brain reserve might consist of developmental and genetic factors such as the total intracranial volume and the apoE gene alleles, and the extent of normal aging changes and brain pathology as previous strokes, atrophy or leukoaraiosis. In terms of lesion load, a small lesion of the association cortex may be adequately compensated by the perilesional cortex, whereas a primary motor cortex bigger area of infarction may need a shift in the interhemispheric lateralization or recruitment of secondary functional centers (Umarova, 2017).

Finally, we have not included other techniques used to measure FC changes post-stroke, such as task-based fMRI and magnetoencephalography, due to space constraints. Soleimani et al. evaluated neural activity using magnetoencephalography longitudinally in four minor stroke survivors, finding correlation between the development of inter-hemispheric connections 6 and 12 months post-stroke and the cognitive recovery of the patients (Soleimani et al., 2023). Shaphaan et al. investigated medial temporal lobe functions post-stroke using the n-back working memory task, which activates the bilateral prefrontal cortex, parietal cortex, anterior cingulate and bilateral cerebellum (Snaphaan et al., 2009). They found reduced medial temporal lobe functionality in post-stroke patients with reduced episodic memory function 6–8 weeks after the event. Further clarification of the pathophysiology behind PSCI, combined with research utilizing different region-selective therapeutic approaches, could lead to significant advances in the field.

Understanding the functional alterations associated with PSCI can be applied to clinical practice to enhance cognitive outcomes after a stroke. Li et al. studied the impact of repetitive magnetic stimulation in the rehabilitation of PSCI using resting-state fMRI (Li et al., 2020a, b). Improvements in cognition and increased neural activity in several cognition-related areas among the intervention group indicate the potential benefits of this therapy. Further clarification of the pathophysiology underlying PSCI, along with research utilizing region-selective therapeutic approaches, could lead to significant advances in the field.

The main limitations of this systematic review were the lack of a biased analysis of the studies selected and the heterogeneity of the neuroimaging protocols, populations and results obtained by each author. Future studies utilizing resting-state fMRI to investigate PSCI should address several key gaps. One significant limitation is the lack of blinding for neuropsychologists conducting cognitive assessments, which can introduce bias. Additionally, there should be more longitudinal studies to capture cognitive changes over time, as only three studies in this review focus on the chronic phase. Exploring specific cognitive domains could facilitate more detailed association analyses between neurofunctional changes and particular cognitive deficits, ultimately enhancing our understanding of PSCI.

## Conclusion

PSCI is a frequent and highly disabling consequence of stroke. Decreased resting-state FC has been observed in several brain areas, including the medial prefrontal cortex, inferior frontal gyrus, and hippocampus, which may contribute to cognitive deficits after stroke. The DMN, encompassing many of these structures, is commonly affected. Additionally, increased FC in contralesional areas, such as the ventral anterior nucleus of the thalamus and middle frontal gyrus, may indicate maladaptation or compensatory changes, particularly within the first three months post-stroke. While resting-state fMRI shows promise in elucidating the mechanisms underlying PSCI, the existing research reveals significant limitations, including study heterogeneity, small sample sizes, and inconsistencies in data processing. We aim to emphasize the importance of rigorous study design and specific inclusion criteria, such as the timing of imaging post-stroke and the location of the infarct. Addressing the limitations identified in this review and employing standardized data processing protocols can facilitate more robust findings in future studies.

## Supplementary Information

Below is the link to the electronic supplementary material.

Supplementary file1 (DOCX 16 KB)

## Data Availability

No datasets were generated or analysed during the current study.
