# Supplementary material for: Resting-state functional connectivity alterations in post-stroke cognitive impairment: a systematic review
Source: Brain Imaging Behav. 2025 Jun 4;19(5):1117–31. doi: 10.1007/s11682-025-01013-w (PMC12518469; doi:10.1007/s11682-025-01013-w)
Supplement: Supplementary file 1 — Supplementary file1 (DOCX 16 KB) [file 11682_2025_1013_MOESM1_ESM.docx]

**Resting-state functional connectivity alterations in post-stroke cognitive impairment: A systematic review**

**SUPPLEMENTARY MATERIAL**

**Supplementary Table 1.** ROBINS-I scale for the studies included in the systematic review

| Study | Missing data | Outcome measurement | Reported results selection |
| --- | --- | --- | --- |
| Ding X et al 2014 | L | M | L |
| Park J et al 2014 | L | M | L |
| Liu J et al 2014 | L | M | L |
| Liu J et al 2017 | L | M | L |
| Dacosta-Aguayo R et al 2014 | L | M | L |
| Dacosta-Aguayo R et al 2015 | L | M | L |
| Siegel JS et al 2016 | L | L | L |
| Peng C et al 2016 | L | M | L |
| Kliper E et al 2016 | L | L | L |
| Bournonville C et al 2016 | L | L | L |
| Lopes R et al 2021 | L | L | L |
| Zang J et al 2020 | L | M | L |
| Vicentini JE et al 2021 | L | L | L |
| Miao G et al 2022 | L | M | L |
| Rao B et al 2022 | L | M | L |
| Zhao Z et al 2021 | L | L | L |
| Jung J et al 2021 | L | M | L |
| Cai H et al 2021 | L | L | L |
| Wang S et al 2022 | L | M | L |
| Min Y et al 2023 | L | M | L |
| Zhu Y et al 2023 | L | M | L |
| Zhao Y et al 2023 | L | M | L |
| Yue X et al 2023 | L | M | L |

*L: Low risk, M: Moderate risk, N: No information, H: High risk.*
